# Identifying Factors Related to Food Agency: Cooking Habits in the Spanish Adult Population—A Cross-Sectional Study

**DOI:** 10.3390/nu10020217

**Published:** 2018-02-15

**Authors:** Ángela García-González, María Achón, Elena Alonso-Aperte, Gregorio Varela-Moreiras

**Affiliations:** 1 Department of Pharmaceutical and Health Sciences, Faculty of Pharmacy, CEU San Pablo University, 28668 Boadilla del Monte, Madrid, Spain; angargon@ceu.es (A.G.-G.); achontu@ceu.es (M.A.); eaperte@ceu.es (E.A.-A.); 2 Spanish Nutrition Foundation (FEN), 28010 Madrid, Spain

**Keywords:** cooking skills, Spain, food literacy, food agency

## Abstract

This study focuses on understanding factors that influence food agency in the Spanish population, specifically with regard to cooking habits, knowledge, and determinants and their possible relationship with body weight. A cross-sectional telephone survey was conducted. Individuals were asked about their cooking responsibilities, how they learned to cook, factors that affect their food choices, and their preferred cooking techniques. Anthropometric data were also recorded. Participants were randomly selected, and we finally had 2026 respondents aged ≥18 years (60% women, 40% men). A total of 90.5% of participants stated that they had cooking skills. Women were mainly responsible for cooking tasks (*p* < 0.05) at all ages. A significantly higher proportion of people under 50 years self-reported that they were “able to cook” in comparison with groups over 50 years. Regardless of age, most participants learned to cook either by practice (43.3%) or from a family member (42.2%). Men tended to be more autodidactic, whereas women reported learning from family. No relation was found between weight status and the evaluated factors investigated. In conclusion, women bear the responsibility for the entire cooking process in families, indicating a gender gap in the involvement of men in cooking responsibilities and competence. More research is needed to assess the influence of cooking knowledge on obesity prevention.

## 1. Introduction

According to the World Health Organization (WHO), poor diet is the main risk factor for early death across the world, especially in Europe [1]. The 2015 Global Burden of Diseases, Injuries, and Risk Factors (GBD) Study, which assessed behavioral, environmental, occupational, and metabolic risk, showed that a high body mass index (BMI) and fasting plasma glucose contributed the most to attributable disability-adjusted life years (DALYs) and that dietary risks accounted for 12.2% of total DALYs for men and 9.0% of total DALYs for women [2]. Energy imbalance and related excess body weight, increased intake of saturated and trans fats, sugar, and salt, and low consumption of vegetables, fruits and whole grains are risk factors for non-communicable diseases in Western countries, and a primary concern for public health institutions [3,4,5].

There is a growing body of recent evidence linking home cooking with healthier dietary choices and better adherence to nutritional guidelines. In fact, the most recent food pyramids, such as the Mediterranean Diet Pyramid and the one designed for the Spanish population [6,7], include culinary skills at the first step, as important tools to achieving a better and healthy diet. Research shows that those who cook and eat more frequently at home usually have a higher intake of fruits, vegetables, and whole grains [8,9,10,11], and an increased amount of time spent on cooking has been linked to lower BMI [12]. On the contrary, eating outside of the home has been associated with increased consumption of ultra-processed foods, ready-to-eat meals, and calorie-dense convenient foods—behaviors all potentially linked to obesity [13,14].

It is remarkable that, while learning cooking skills and gastronomy is becoming fashionable in social and mass media, there is a concurrent decline in home cooking worldwide [15,16,17]. The decrease in home cooking can be explained by a plethora of reasons, including urbanization, work schedules, and the incorporation of women into the workforce—since cooking has traditionally been women’s responsibility [11,18]. Currently, whilst cooking is in principle no longer needed to feed oneself, the absence of cooking skills and competence can be considered a public health problem, since not knowing how to cook is a barrier to healthy food preparation [19].

Community interventions have been demonstrated to be effective in improving the cooking skills of participants, when cooking skills are defined as “the mechanistic and physical skills that are applied during food preparation” [20]. However, their effectiveness for improving healthy eating is more inconsistent [10,21,22]. Knowledge is necessary, but may not be enough to trigger behavioral changes. An individual may be able to chop vegetables, roast a chicken, or follow a recipe, but be unable to design a meal or take the decision of what to eat and when. Recently, three similar concepts have emerged in literature: cooking competence, food literacy, and food agency [23,24,25]. These three terms have shifted the focus from the traditional technical-centered approach to a wider concept that explains how individuals use nutritional knowledge and cooking skills to prepare a meal within a particular food environment. The new approach considers socio-economic aspects as planning, budgeting, time, mobility, storing, eating, and waste disposal. People with food agency are empowered not only to cook but to prepare healthy meals and to improve their nutritional status [24]. Thus, interventions aimed at improving people’s food agency can empower a population to eat healthily.

To design effective educational intervention projects, it is essential to know which factors influence food agency [26]. From an ecological, multilevel perspective, food agency depends on intrapersonal factors (e.g., age, sex, beliefs, knowledge, and attitudes), interpersonal factors (e.g., family and friends), and community factors (e.g., food availability, prices, and mass media) [27,28]. Our research group has previously shown that personal, socioeconomic, and cultural factors currently influence food purchasing in the Spanish population [29]. The present study focuses on understanding some of the factors that influence food agency—considered to be the entire culinary process—for a representative sample of the Spanish population. The relationship of these factors to body weight is also investigated, under the premise that eating and cooking at home is not solely about having cooking skills, but a holistic issue embedded in a particular social, cultural, anthropological, and economic environment.

## 2. Materials and Methods

### 2.1. Sample and Study Design

This study was designed as a cross-sectional survey of a nationally representative sample of adults (≥18 years old) living in Spain. Details on the sample, methods, and study design have been published elsewhere [29].

In summary, a questionnaire of 50 items was specifically developed for this study: 16 items focused on sociodemographic aspects, 8 focused on food eating patterns, 13 focused on shopping habits, 9 focused on cooking habits and skills, and 4 focused on nutrition knowledge and perception (see Appendix A).

The questionnaire was designed after careful analysis of literature on food habits in Spain [30,31,32]. It was face-validated by experts from the Consulting Agency that performed the survey and had previously participated in surveys on food habits in Spain. Some changes to adapt options and questions were made following their feedback. The survey was completed by individuals in private dwellings and was randomly administered by telephone by professionally trained surveyors via computer-assisted telephone interviewing (CATI). This allowed automation of the process and minimized the non-response rate. Only fixed-line phone numbers were used. To avoid bias derived from the unequal presence at home due to work situation, age, or sex, participants were contacted at several different times between 1:00 and 9:30 p.m. until a response was obtained. Questionnaires with more than 60% of blank questions were rejected for analysis. Participants resided in all territories of Spain, except for the Canary Islands and the Autonomous Cities of Ceuta and Melilla in North Africa. Sampling was stratified and a weighting factor was applied in order to ensure the representativeness of all strata. 

For data analysis, participants were categorized into the following age groups: 18–30, 31–49, 50–64, 65–75, and >75 years, following census data published by The Spanish National Statistics Institute [33]. 

Body mass index was calculated from values reported by participants for weight and height, and results were classified in four categories, following the WHO-Europe criteria [34]:

• Underweight: <18.5 kg/m^2^;
• Normal range: 18.50–24.99 kg/m^2^;
• Pre-obesity: 25.00–29.99 kg/m^2^;
• Obesity: ≥30 kg/m^2^.

The study was conducted in accordance with the Declaration of Helsinki, and all data were collected anonymously and recorded according to the Spanish Organic Law of Personal Data Protection (LOPD) 15/1999. Since participants could not be tracked, there was no need for informed written consent. Nonetheless, before the questionnaire was answered, participants were informed about the objective of the study and asked for permission to use and publish the data.

### 2.2. Statistical Analyses

Analysis was performed not taking into account missing values, due to a low non-response rate. For questions with a higher non-response rate, differences in main characteristics (sex, age, education, and so on) of respondents and non-respondents were analyzed, and no statistical difference was found for any of the variables presented in this paper. 

Results for categories are reported using frequencies and percentages, continuous variables are reported using mean ± standard deviation.

For results given as distributions (in terms of cooking ability; how cooking is learned, criteria used when planning meals, and preferred cooking techniques), differences between groups were evaluated using a chi-square test (*z*-test for multiple comparisons). A two-tailed Student’s *t*-test and ANOVA with a Bonferroni post-hoc test were performed to evaluate the differences between sex and age groups when dependent variables were continuous. To control the effect of gender and age while analyzing differences in behaviors, a binary logistic regression model was applied, with “men” and “under 18” as residual categories. For all statistical analysis, differences were considered significant at *p* < 0.05. Statistical analyses were performed using SPSS v.24.0 (IBM Corp., Armonk, NY, USA) and Med Calc, v.17.9 (Med Calc Software, Ostend, Belgium).

## 3. Results

A total of 31,552 phone calls were made, and the participation rate was 6.42%. Once a respondent was enrolled in the survey, the non-response rate to questions was very low (1.5% as an average, and a maximum of 29%). Questionnaires with more than 60% of blank questions were rejected for analysis.

A total of 2026 respondents (1223 women and 803 men) were used for the analysis. The final sampling error was 2% for global data (33), calculated for a 95% level of confidence and probability of positive (*p*) and negative (*q*) responses *p* = *q* = 0.5. In terms of age, 320 respondents were between 18 and 30 years old, 801 were between 31 and 49 years old, 460 were between 50 and 64 years old, 218 were between 65 and 75 years old, and 227 were older than 75 years old. Representativeness error for age distribution and for sex distribution was 2.8%.

### 3.1. Cooking Task: To Cook or Not to Cook

**Figure 1:**
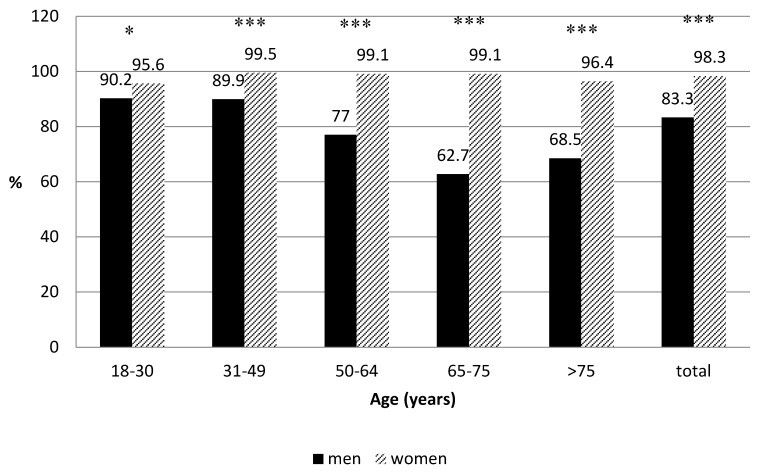
The percentage of survey respondents who stated that they know how to cook. The sample sizes by age groups were as follows: 18–30 years: 131 men and 189 women; 31–49 years: 332 men and 469 women; 50–64 years: 185 men and 275 women; 65–75 years: 84 men and 134 women; >75 years: 71 men and 156 women. * *p* < 0.05; *** *p* < 0.0005, women vs. men.

Of the participants, 90.5% stated they had cooking skills. As shown in Figure 1, the percentage of women that declared that they knew how to cook was statistically higher than in men (98.3% vs. 82.3%, *p* < 0.001), a difference that was seen in all age groups. Once adjusted by sex using a binary logistic regression, the percentage of respondents that stated that they knew how to cook was higher in those under 50 years, compared to those 51 years and over (*p* < 0.05).

**Figure 2:**
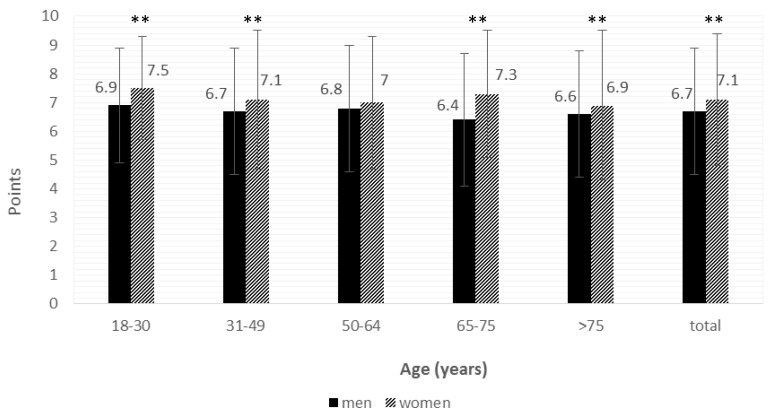
The average population that enjoys cooking, on a scale from 1–10. The sample sizes by age groups were as follows: 18–30 years: 131 men and 189 women; 31–49 years: 332 men and 469 women; 50–64 years: 185 men and 275 women; 65–75 years: 84 men and 134 women; >75 years: 71 men and 156 women. ** *p* < 0.01, women vs. men.

Cooking competence depends not only on having skills but also on the willingness to cook, so participants were also asked to evaluate how much they enjoyed cooking on a scale from 1 to 10. The population mean was 6.95 ± 2.25. As seen in Figure 2, women enjoyed cooking more than men. There were significant differences (*p* < 0.01) between the observed score assigned by men (6.7 ± 2.2) and by women (7.1 ± 2.3) in the entire population. Similar differences were also seen when stratifying by age groups, except for the participants who were 50–64 years old. However, no differences were observed between age groups for this parameter.

Of the entire population, 53.6% reported being responsible for all the cooking at home and 17% reported being responsible for more than 50% of the cooking. In line with the previous outcomes, it seemed logical to find that women reported having the main responsibility for cooking in the family. Therefore, we performed a binary logistic regression model in which the dependent variable took the value “1” if more than 50% of cooking was carried out by that person and “0” if not; the independent variables were sex and age. The results (Table 1) show women had a sevenfold higher probability than men of bearing the responsibility for more than 50% of cooking tasks. Once the impact of sex was controlled, it was observed that those under 30 years took less responsibility for cooking, when compared to other age groups.

The percentage of people that reported they never cooked was 20.2% in men and was notably lower in women, 4.7% (*p* < 0.0005), with the highest percentage for men in the group aged 65–75 years (31.3%), followed by the group aged 18–30 years (26.5%). For both men and women, the age group with the highest percentage of people that do not cook was those aged 18–30 years (12.6%).

As expected, there was a difference in the preferences for cooking between those who had a higher responsibility for cooking and those who did not, the former showing a score in the scale “liking to cook” of 7.2 ± 2.2 vs. 6.5 ± 2.1, (*p* < 0.0005).

Since women were mainly responsible for cooking, it is not surprising that women were found to spend more time cooking per week (9.18 ± 6.5 h/week) than men (7.5 ± 5.4 h/week; *p* < 0.0005). However, interestingly, no differences were found between age groups for this factor. For the entire population, regardless of sex and age, the average time spent cooking was 8.7 ± 6.2 h/week. 

### 3.2. Learning How to Cook

Learning how to cook has traditionally taken place within families, although the current proliferation of cookery books and TV shows makes it reasonable to hypothesize that the manner in which people learn how to cook could be changing. Table 2 shows the different ways that participants in the survey learned how to cook. Those that have never cooked or that did so occasionally were not asked this question, nor were they asked about criteria used when planning a meal.

Regardless of age, most participants had learned to cook either by practicing on their own (43.3%) or from a family member (42.2%). In general, men tended to be more autodidactic, whereas women reported that they had learned from members of their family (32.6% men vs. 46.5% women, *p* < 0.05). The option “others” was more frequent in women, and in both the youngest and oldest groups, and included “friends” and “colleagues” as the most frequent answers. The proportion of people over 50 years who had learned how to cook using the Internet, compared with the youngest age groups, was significantly lower (*p* < 0.05).

### 3.3. Decisions Made While Preparing a Meal

**Figure 3:**
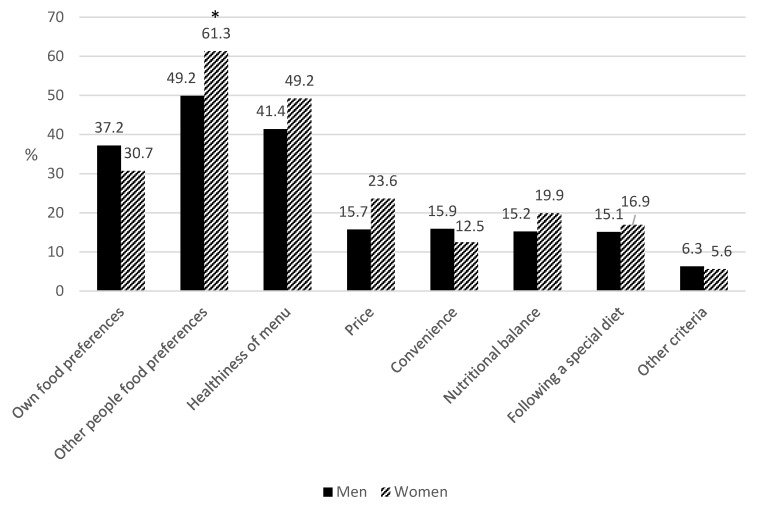
The main criteria when planning meals at home (%), by gender. The sample sizes by gender were as follows: *n* = 406 men; *n* = 890 women; * *p* < 0.05, women vs. men.

Meal planning is a key issue in food agency. In our questionnaire, we asked which factors were the most important when designing a meal. Results in Table 3 and Figure 3 show that, regardless of age, the main criteria while planning meals were “other people’s food preferences,” followed by “own food preferences and dislikes,” and “the healthiness of the menu.” Forty-five persons gave personal answers to the question on “menu planning,” but during the coding process most of them were included in an existing category (i.e., “I cook what my children like” was included in “other people’s preferences”). Some other options included “maintaining variety” (*n* = 7), “it depends on the time I have for cooking” (*n* = 3), and “I adapt menu to food availability in the market” (*n* = 4). 

Differences were found between women and men, since the “other people’s food preferences” criterion was more important for women (*p* < 0.05). In addition, criteria related to “health worries” (e.g., “healthiness of menu” and “nutritional balance”) were more significant for women than for men (*p* < 0.05), as were concerns about price (*p* < 0.05). As regards differences between age groups, “other people’s food preferences” was a more common choice in the groups of people between 31 and 64, and “convenience” was more common in the younger groups.

### 3.4. Cooking Techniques

**Figure 4:**
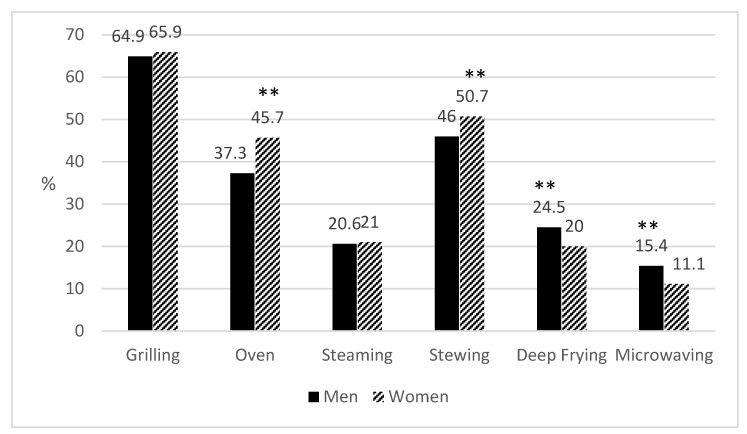
Preferred cooking techniques. Sample sizes by gender: *n* = 792 men; *n* = 1219 women. ** *p* < 0.01, women vs. men.

Finally, the cooking techniques that were the most appreciated and frequently used by the Spanish population were also evaluated. Results are shown in Table 4 and Figure 4.

Thirty-eight percent of the population showed a preference for using just one technique, 36% for two, and only 6% declared using the six possible proposed cooking methods. Over the entire population, the cooking techniques most frequently used were grilling (66%), stewing (49%), and baking (42%). In terms of sex, women used more “slow” preparation techniques, such as baking and stewing (*p* < 0.05), whilst men used more “quick” techniques, such as deep frying and microwaving (*p* < 0.05). In addition, although some statistical differences were found between age groups, they seemed to be spurious, so we concluded that age does not have an influence on the choice of cooking techniques.

### 3.5. Results by Weight Groups

Ninety percent of the respondents declared their weight and height, and BMI was calculated. Most of the participants (51%) were in the normal range of weight, only 1% presented as underweight, 33% of the population showed pre-obesity values, and 9% were considered obese. There was a significantly higher prevalence of being underweight in women than in men (2.8% vs. 0.3%; *p* < 0.05), whereas the prevalence of pre-obesity in men was significantly higher than in women (42.1% vs. 23.8%, *p* < 0.05). Differences in prevalence by age groups were found between the younger group and the rest of the age groups; both underweight (5%) and normal-weight (65%) were more prevalent in the group aged 18–30 years, compared to other age groups (*p* < 0.05).

Once the effect of sex and age was controlled, no differences were observed for the BMI according to whether they knew how to cook or not, whether they enjoy or dislike cooking, nor the time spent in this task. Statistical significance was only seen in cooking responsibility. Those people classified as underweight were usually more frequently responsible for less than half of the cooking at their homes (*p* < 0.05) when compared to obese volunteers; however, no relation to other BMI groups was observed. 

Table 5 shows the factors that may influence cooking competence according to BMI. Results were similar to findings for the entire population and no significant differences were found in the declared way of learning how to cook or in preferred cooking techniques. Regarding the criteria used to plan meals at home, underweight and obese groups consider their own food preferences in a higher proportion to people within a normal range of weight or overweight people (*p* < 0.05). 

## 4. Discussion

There is growing interest in cooking and food preparation skills across the population and within specific subgroups, and in the implications they may have on food choice and overall health. Previous research has linked home cooking with healthier diets [10,11], and most obesity prevention programs include cooking skill interventions as a tool to empower people to be able to maintain a healthy weight [35,36,37,38]. Nevertheless, the impact of those cooking lessons on behavioral changes or weight status is a controversial topic. Short-term changes in health behavior have been observed after cooking lessons [39,40], but long-term results are not so common. The EAT Project examined involvement in food preparation over time in adolescents (15–18 years), emerging adults (19–23 years), and those in their mid-to-late twenties (24–28 years) with a 10-year follow up, finding that associations between adolescent food preparation and dietary factors during the mid-to-late twenties were largely null [39]. Along the same line, a recent review that evaluated cooking and food skill interventions from across the globe reported that only 14 out of 59 had long-term effects [41].

Both the Mediterranean Pyramid and the Dietary Guidelines for the Spanish population have been updated to introduce recommendations of methods of selecting, cooking, and eating foods, together with proportion and frequency recommendations for consumption of each food group. “Cooking” is now part of the basic level of the food pyramid and is thus considered an important activity that requires the proper time and space, and using appropriate techniques that provide a healthy, safe, and tasty diet [6,7].

Some literature describes factors that influence home cooking in Canada, the United States, and the United Kingdom [42,43,44], but scarce research has been done on cooking competence of the general population in Spain or, more globally, in the Mediterranean area [45,46]. Knowing peoples’ attitudes towards cooking as well as the factors that impact the act of cooking in the population is an essential requirement when designing evidence-based educational programs. Healthy cooking is a complex activity [47] and thus it is difficult to understand the influencing factors, but changing one’s health behavior can lead to changes in others [48], so it is an important topic for research. 

### 4.1. To Cook or Not to Cook

The ability to cook does not seem to be a problem in Spain, as a high percentage of the participants in our survey, roughly 90%, stated they were able to cook, although only 70% were responsible for more than 50% of cooking tasks at home. Our results also show that, presently, cooking in Spain is still primarily carried out by women. Regardless of age, women reported being able to cook in higher proportions than men, more often reported being the person responsible for cooking at home, and reported spending more time cooking than men. They also enjoyed cooking more than men. These results follow the same pattern observed in previous studies from other countries, where women are primarily responsible for food preparation functions, and generally report higher self-confidence or efficacy with cooking and food preparation skills compared to men [15,18,44,49]. 

It is important to notice that the difference between the percentage of women who cook, compared to men, is significantly lower in the younger population than in participants over 50 years old. In fact, the difference between the number of men and women that declared cooking skills was 5% in the group between 18 and 30 years of age, but 9.6% in the participants aged 31 to 49 years, 22% in the group aged 50 to 65 years, 36% in those between 65 and 75 years, and 27% in the oldest group. Therefore, cooking competence is increasing in men in younger generations.

Furthermore, the incorporation of men in cooking tasks in the youngest groups added to the result in a significantly higher proportion of young people (male and female) that self-reported to be “capable of cooking,” in comparison with groups over 50 years old. These results differ from other published studies, which report that the young are less confident in cooking than older adults [22,43,44]; however, none of these studies were carried out in Spain, and they used different methodologies to assess culinary skills. There are few studies on cooking skills in young people in Spain. In Barcelona, Sainz Garcia [45] measured different aspects related to cooking using objective tools on a sample of university students (18–24 years) and found that 55% of the participants felt “very confident” with the cooking skills analyzed—a percentage considerably lower than the one we report here. Thus, we may conclude that the younger participants in our survey (18–30 years), both men and women, are more familiar with cooking skills than other similar populations in comparable studies.

Young people’s interest in cooking, and food in general, is obvious from the proliferation of mass-media shows and the amount of information about food and recipes that appears on social media. In 2014, 177 million pictures were uploaded to Instagram using the hashtag “food,” and 63% of people under 32 years old published a picture of their own food or beverage on social media. Some opinion surveys conducted by consulting agencies [50,51] researched interest in food and cooking in so-called Millennials (people born between the 1980s and 2000s), revealing a clear interest in cooking and food not seen in previous generations, such as “Generation Xers” (those born between 1970s and 1980s) or the baby-boomers (those born in the 1960s). The Millennials correspond with our younger age group (18 to 30 years).

Nonetheless, being interested in food and recipes, although an important point, is not enough to provide cooking skills and does not imply being responsible for cooking or having cooking competence. In our study, people under 30 years old had less responsibility for cooking tasks, when compared to any other age group, and the highest percentage of people that had never cooked was also seen in this age group. Similar results have been reported in Canada, where young adults report minimal involvement in food purchasing and preparation activities, despite indicating that their skills and resources were adequate [44]. Self-reported cooking skills may be unrelated to everyday cooking [52]. Individuals can decide not to cook at home because another household member takes responsibility for this, they may eat elsewhere, or they may not prioritize time for cooking. Similarly, the low participation in cooking tasks of the youngest group is in accordance with our previously published results on food shopping habits in Spain, with young adults (18–30 years old) being the least likely to be responsible for food shopping [29]. Both findings support the hypothesis that the low food preparation enrollment of people in this age group could be due to the delayed age in leaving the family household in Spain since, according to statistics, only 19% of the population between 16 and 29 years live independently [53]. Efforts should be made to include young people in cooking tasks as soon as possible, because some literature shows that the younger the person starts having cooking responsibilities, the healthier their diet throughout their lifespan will be [54].

Women were found to enjoy cooking more than men, which is in line with other studies [55], and there was no difference across age groups. Enjoying cooking, together with the fact that women are usually the person responsible for cooking and that they use slower cooking techniques, may explain why women spend more time cooking than men, a result also seen in other studies [55].

### 4.2. How People Learn to Cook

While literature shows that the mother or any other member of the family has been consistently identified as the primary role model and teacher of cooking skills [54,56], our study showed a more even distribution of the percentage of the population that learned to cook from the family (42.2%) and those who learned how to cook through self-experience (43.3%). Furthermore, a previously unreported gender difference in the way of learning how to cook was found. Learning from a family member was the most usual way to acquire cooking skills for women, but men declared themselves more often self-taught and to have learned cookery skills through self-experience. Wolfson et al. [57], in a recently published study on how people from the United States learned to cook (based on interviews with focus groups and a nationally representative survey), also found a significantly higher percent of women who learned how to cook from their parents (72% vs. 61%, *p* < 0.001); however, they did not find differences by gender in the group who taught themselves to cook.

Research shows that men and women learn and perceive in a different way, females being more interpersonally oriented, while males are more task-centered [58]. López-Torres defines two phases in the culinary learning process: the “transmission phase” and the “learning phase.” In the first phase, the learners acquire knowledge and skills in a passive way, unconsciously by socialization, observing, helping, and talking with the cookers around them. In the second phase, the learners search for information in an active way, asking, reading, practicing, and learning by trial and error [59]. The recalled data in our survey do not give us enough information to explain the observed gender difference. Nonetheless, we can hypothesize that, since cooking has been traditionally considered a woman’s task, girls are more prone to pay attention to their mother’s or any other family member’s cooking, and to learn from them, while men start only after they “need to do it” because no other person prepares their meal.

Although TV cooking shows are increasing in number and audiences may reach up to 25% of audience in Spain [60,61], the percentage of the population that stated they had learned how to cook from TV or books is still low (3–7%). Therefore, in line with other studies [57], it seems that TV cooking shows are considered only as entertainment by most of the population and have no or little influence on peoples’ culinary skills.

In accordance with previous studies [57], we did not find any difference in the way people claimed to have learned how to cook by age group, although the Internet as a learning tool is more frequent in people under 50 years old. These results are in line with the data on the use of ICTs (Information and Communication Technologies) by the Spanish population: although increasing in number, only 24% of Internet users in Spain are over 55 years [62]. 

In relation to general skills acquisition, the most effective time for learning new skills is from childhood to early adolescence [63]. In Spain, since the year 1990, food and nutrition knowledge has been included in the school curriculum, following the recommendations of the Dublin European Conference of Education for the Health of the European Community [64]. However, cookery lessons have never been part of Spanish school curricula, thus leaving learning cooking skills as a private matter. Under the umbrella of the Governmental NAOS strategy (“Nutrición, Actividad Física y Prevención de la Obesidad” = Strategy for Nutrition, Physical Activity and the Prevention of Obesity) [65], whose goal is to reverse the trend in obesity prevalence through the promotion of healthy diets and physical exercise, some interventional programs that include cookery lessons have been run, showing success in changing health behaviors in the short term [66]. The Food, Nutrition and Gastronomy Program for Pre-School Education—PANGEI, “It’s My Pleasure” [67]—was designed by the Spanish Ministry of Health together with the National Centre for Educational Innovation and Research (CNIIE), the Food and Nutrition Safety Agency, the Royal Academy of Gastronomy, and the Spanish Nutrition Foundation in order to help young children (preschool-aged) to acquire healthy eating habits from an early age. The project comprises updated innovative material, with a gastronomic approach that includes cookery lessons, aimed at improving the food culture in Spain. PANGEI encourages young children to develop a taste for food, facilitating an ability to taste, smell, and appreciate the texture of food, as well as reinforcing the ideas that kitchens are a commonplace area where all members of the family can and must participate and that the meals are not an individual event but a very important social element that has to be valued [67]. However, follow-up research should be done to determine the longer-term impact of the project.

### 4.3. Cooking Techniques

Analyzing the most appreciated cooking techniques as declared by the participants may give us some information on population cooking confidence and knowledge of healthy cooking. The most preferred method in the population was “grilling” (66%), a method commonly linked to healthy diets [7]. Stewing and baking were also chosen by a large percent of the population, both being common to traditional gastronomical culture in Spain. In the analysis by subpopulations, no clear differences were found by age group, but some gender differences were found: women use more slow and skillful preparation techniques such as baking or stewing, while men use quicker and easier techniques, such as deep frying and microwaving. These results are comparable to others previously published [43] and are in line with the finding that women learn how to cook from a family member and so are more attached to traditional cooking methods and recipes.

### 4.4. Preparing a Meal

Research shows that a significant percentage of the population are satisfied with their self-perceived cooking and food preparation skills, although the actual use of these skills is highly variable, since many other factors also affect the activity of cooking. These factors depend not only on skills, but also on knowledge, attitudes, and sociocultural factors. To determine the culinary competence and food agency of a population, it is important to know what factors determine what food is selected for preparation [19,24,42]. On this matter, taste, nutritional value, cost, and time have been described as the primary factors influencing preparation decisions. Our results match others found in studies upon Spanish and non-Spanish populations [44,59,68], showing a general framework for “decision-making” when planning meals, in spite of different environmental and food cultures, and highlighting the importance of the sociability of eating and the pleasure of sharing a meal. In this area, some gender differences were found; for example, women were more concerned than men by other people’s food preferences, the healthiness of the meal, and the costs. These results agree with previously published results by our research group [29] and those mentioned in Section 4.1, both showing women as the member of the family in charge of cooking tasks at home and in charge of most food shopping in Spain.

### 4.5. Results by BMI Categories

Maintaining a healthy weight depends upon, besides other important factors, an individual’s ability to use healthy ingredients and techniques. Understanding the relationship between cooking skills and weight is important, given that meal preparation at home is increasingly being promoted as an obesity reduction complimentary tool [10]. Conversely, our results do not show any relation between population BMI and having cooking skills, cooking frequency, time spent in cooking, or any of the other analyzed factors. 

Many studies have found a relationship between cooking skills and interest, and healthier food choices [10,37,44]. However, like ours, some other studies failed to find a direct correlation between cooking skills, cooking frequency, and body weight [37,46]. These apparently contradictory results reflect the fact that having cooking competence, while important to be able to cook in a healthy way when trying to lose weight [37], does not seem to be enough to prevent weight gain. It is essential to emphasize the need of further studies to understand the relationship between cooking and body weight. 

In our results, 42% of the population reported an excess of weight. We found 33% of pre-obesity, a percentage similar to the recently published national data of the ENPE study (Estudio Nutricional en Población Española = Nutritional Study for the Spanish Population) [69] that showed a pre-obesity rate of 39%. However, it is important to note that only 9% of our surveyed population reported obesity, while in the ENPE study this percentage was 22%. Other research that analyzed self-reported weight, published by the Organization for Economic Co-operation and Development (OECD) [70], showed an intermediate value of 16% obesity in Spain. A recent report by the Spanish Society for the Study of Obesity (SEEDO) showed that 82.2% of obese people do not recognize themselves as obese and 12% think they are at a normal weight. Thus, although relevant because of the high representativeness of the sample, our results linking cooking aspects and weight status should be carefully interpreted because of the potential underreporting of weight bias.

## 5. Conclusions

Our results show that women are mainly responsible for the entire cooking process in the family, being the main transmitters of cooking skills and traditional gastronomic techniques for future generations. Men are slowly being incorporated into cooking, but there is still an important gender gap when studying the involvement of men in cooking responsibilities and competence. 

Finally, no relation has been found between weight status and different aspects of culinary process. More research is needed on the influence of cooking knowledge and obesity prevention.

## 6. Strengths and Limitations

The sample size and its representativeness of the entire country area is an important strength of our study, but some important limitations should be mentioned. Results are expressed on a cross-sectional basis and thus do not reflect a possible evolution during a specific period of time. Only home phones were used; although such phones are present in 78% of homes in Spain, this could have limited access to some parts of the population. Home phones were chosen because, although availability is more likely when calling mobile phones, collaboration is lower and can introduce greater biases, since it is more difficult to answer the questionnaire when people are at work or otherwise occupied. It is important to take into account the duration of the questionnaire (more than 20 min in this case), which requires a degree of dedication and attention. Therefore, we decided it was better to assume that errors of bias derived from the total non-penetration of the home telephone instead of from the unequal collaboration dependent on the type of telephone used. Furthermore, by calling the landline, distractions are avoided, and the quality of the answers to the questionnaire improves. Data were self-reported and therefore may have suffered from memory, response, or social desirability bias. Other limitations include the low participation rate—despite the fact that we found a good level of involvement in the survey—and the fact that validation of the survey was only based on face validity.

## Figures and Tables

**Table 1:** Binary logistic regression for the main cooking responsibilities.

	Odds Ratio	*p*	CI (95%)	
Gender *				
Women ^a^	7.25	<0.0005	5.704	9.213
Age ** (years)				
31–49 ^b^	4.044	<0.0005	2.945	5.554
50–64 ^b^	3.499	<0.0005	2.453	4.992
65–75 ^b^	3.822	<0.0005	2.408	6.068
>75 ^b^	3.753	<0.0005	2.381	5.914

* Sample size by gender: men: 803; women: 1,223. ** Sample size by age groups; 18–30 years: *n* = 320; 31–49 years: *n* = 801; 50–64 years: *n* = 460; 65–75 years: *n* = 218; >75 years: *n* = 227. ^a^ Compared to men. ^b^ Compared to 18–30 years. CI: Confidence interval

**Table 2:** Reported methods by which cooking was learned (%) by age and sex.

	18–30 Years			31–49 Years			50–64 Years			65–75 Years			>75 Years		
	Men (*n* = 59)	Women (*n* = 83)	Total (*n* = 142)	Men (*n* = 208)	Women (*n* = 354)	Total (*n* = 562)	Men (*n* = 83)	Women (*n* = 216)	Total (*n* = 299)	Men (*n* = 26)	Women (*n* = 113)	Total (*n* = 139)	Men (*n* = 30)	Women (*n* = 124)	Total (*n* = 154)
Own experience	54.2 ^1^	30.1 *^,1^	40.1 ^1^	51 ^1^	38.4 *^,1^	43.1 ^1^	60.4 ^1^	43.1 *^,1^	47.8 ^1^	42.3 ^1^	44.2 ^1^	43.9 ^1^	56.7 ^1^	32.3 *^,1^	37 ^1^
From family	39 ^1^	48.2 ^2^	44.4 ^1^	32.2 ^2^	46.6 *^,2^	41.3 ^1^	26.5 ^2^	45.8 *^,1^	40.5 ^1^	42.3 ^1^	45.1 ^1^	44.6 ^1^	33.3 ^1^	48.4 *^,2^	45.5 ^1^
Books/TV	0	4.8 ^3^	2.8 ^2^	2.4 ^3^	3.7 ^3^	3.2 ^2^	3.6 ^3^	3.7 ^2^	3.7 ^2^	3.8 ^2^	5.3 ^2^	5 ^2^	10 ^2^	6.4 ^3^	7.1 ^2^
Internet	1.7 ^2^	4.8 ^3^	3.5 ^2^	4.8 ^3^	2.8 ^3^	3.6 ^2^	2.4 ^3^	0	0.7 ^2^	0	0.9 ^2^	0.7 ^2^	0	0	0
Cooking courses	5.1 ^2^	2.4 ^3^	3.5 ^2^	1 ^3^	1.1 ^3^	1.1 ^3^	2.4 ^3^	0.9 ^2^	1.3 ^2^	7.7 ^2^	0.9 ^2^	2.2 ^2^	0	0.8 ^4^	0.6 ^2^
Other	0	9.7 *^,3^	5.7 ^2^	8.6 ^4^	7.4 ^4^	7.7 ^4^	4.9 ^3^	6.5 ^2^	6 ^2^	3.9 ^2^	3.6 ^2^	3.6 ^2^	0	12.1 *^,3^	9.8 ^2^

* *p* < 0.05, women vs. men. Different superscripts show statistical differences (*p* < 0.05) within the same column. Non-respondents: *n* = 307; *n* = 423 were not asked this question.

**Table 3:** Main criteria used when planning meals at home (%) by age and sex (multiple choice question).

	18–30 Years			31–49 Years			50–64 Years			65–75 Years			>75 Years		
	Men (*n* = 59)	Women (*n* = 83)	Total (*n* = 142)	Men (*n* = 208)	Women (*n* = 354)	Total (*n* = 562)	Men (*n* = 83)	Women (*n* = 216)	Total (*n* = 219)	Men (*n* = 26)	Women (*n* = 113)	Total (*n* = 139)	Men (*n* = 30)	Women (*n* = 124)	Total (*n* = 154)
Own food preferences	32.8 ^1^	44.2 ^1^	39.5 ^1,a^	28.2 ^1^	29.5 ^1^	29 ^1,b^	38.4 ^1^	27 *^,1^	30.1 ^1,b^	45.6 ^1^	26.9 ^1^	32.8 ^1,a,b^	40.4 ^1^	32 ^1^	33.7 ^1,a,b^
Other people’s food preferences	46.3 ^1^	56.5 ^1^	52.3 ^2,a^	53.2 ^2^	66.2 *^,2^	61.4 ^2,b^	50 ^1^	65.6 *^,2^	61.4 ^2,b^	37.7 ^1,b^	55.7 ^2^	52.4 ^2,a^	44.6 ^1,a^	47.9 ^2^	47.2 ^2,a^
Healthiness of menu	33.6 ^1^	47.8 ^1^	41.9 ^1,a^	43.3 ^3^	51.3 ^3^	48.3 ^3,a^	41.4 ^1^	46.8 ^3^	45.3 ^3,a^	51.4 ^1^	49.6 ^2^	49.9 ^2,a^	34.4 ^1^	48.1 ^2^	45.4 ^2,a^
Price	12.2 ^2^	23.2 ^2^	18.7 ^3,a^	14.6 ^4^	23.3 *^,1^	20 ^4,a^	24.3 ^2^	21.6 ^4^	22.6 ^4,a^	3.3 ^2^	25.7 *^,1^	21.8 ^3,a^	16.2 ^2^	26.3 ^1^	24.3 ^1,a^
Convenience	13.5 ^2^	21 ^2^	17.9 ^3,a^	15.2 ^4^	14.8 ^4^	14.9 ^5,a^	23.7 ^2^	11 *^,5^	14.4 ^5,a^	0	8.8 ^3,4^	7.24 ^5,b^	6 ^2^	6 ^3^	8.4 ^3,b^
Nutritional balance	8.3 ^2^	19.8 ^2,3^	15.2 ^3,4,a^	16.5 ^4^	23.8 *^,1^	21.1 ^4,a^	24.2 ^2^	20.4 ^4^	21.5 ^4,a^	0	12.6 ^3^	10.3 ^4,a^	7 ^2^	14.^5,4^	13 ^3,a^
Following a special diet	17.2 ^1,2^	20.6 ^2^	19.2 ^3,a^	14.1 ^4^	17.3 ^4^	16.1 ^4,a^	19.9 ^2^	17.9 ^4^	18.5 ^4,a^	10.5 ^2^	16.7 ^1,3^	15.5 ^3,4,a^	8.9 ^2^	11.9 ^3,4,5^	11.3 ^3,a^
Other criteria	6.9 ^2^	9.8 ^3^	8.6 ^4,a^	6 ^5^	7 ^5^	6.6 ^5,a^	4.6 ^3^	4.6 ^6^	6.2 ^6,a^	1.9 ^2^	4.2 ^4^	3.8 ^5,a^	0	2.5 ^6^	2.6 ^4,a^

* *p* < 0.05, women vs. men. Different numbered superscripts show statistical differences (*p* < 0.05) within the same column (differences intra-group for meal planning criteria). Different lettered subscripts show statistical differences (*p* < 0.05) between age groups. Non-respondents: *n* = 307; 423 respondents were not asked this question.

**Table 4:** Preferred cooking techniques by age and sex (%).

	18–30 Years			31–49 Years			50–64 Years			65–75 Years			>75 Years		
	Men (*n* = 162)	Women (*n* = 157)	Total (*n* = 319)	Men (*n* = 404)	Women (*n* = 393)	Total (*n* = 797)	Men (*n* = 224)	Women (*n* = 232)	Total (*n* = 456)	Men (*n* = 102)	Women (*n* = 116)	Total (*n* = 218)	Men (*n* = 85)	Women (*n* = 136)	Total (*n* = 221)
Grill	59.8 ^1^	71 *^,^^1^	65.5 ^1,a^	68.7 ^1^	61.5 *^,1^	65.2 ^1,a^	68.4 ^1^	72.2 ^1^	70.4 ^1,b^	61.4 ^1^	62.2 ^1^	61.8 ^1,a^	57.3 ^1^	67.4 ^1^	63.8 ^1,a^
Oven	30.4 ^2^	40 ^2^	35.1 ^2,a^	46.9 ^2^	53.1 ^2^	44.9 ^2,b^	38.7 ^2^	56.6 *^,2^	47.8 ^2,b^	28.7 ^2^	38.9 ^2^	34 ^2,a^	40.6 ^2^	33.8 ^2^	36.4 ^2,a^
Steaming	12.5 ^3^	15.9 ^3^	14.2 ^3,a^	22.8 ^3^	18.4 ^3,4^	20.6 ^3,a^	20.3 ^3^	29.7 *^,3^	25.1 ^3,b^	23.4 ^2^	16.3 ^3^	19.6 ^3,a^	25.3 ^3^	24.4 ^2,3^	24.7 ^3,b^
Stew	35.1 ^2^	23.7 *^,3^	29.5 ^2,a^	40.3 ^4^	49.7 *^,2^	45 ^2,b^	53.6 ^4^	57.8 ^2^	55.7 ^4,c^	62.2 ^1^	65.4 ^1^	63.9 ^1,d^	59.3 ^1^	62.1 ^1^	61 ^1,c,d^
Frying	27.4 ^2^	16.2 *^,3^	21.9 ^4,a^	22.7 ^3^	20.3 ^3^	21.5 ^3,a^	26.3 ^5^	22.3 ^3^	24.3 ^3,a^	22.4 ^2^	17.3 ^3^	19.7 ^3,a^	27.5 ^2,3^	23.1 ^2,3^	24.8 ^3,a^
Microwave	12 ^3^	7.7 ^4^	9.8 ^3,a^	7.9 ^5^	9.3 ^4^	12.4 ^4,a^	13.6 ^6^	12.3 ^4^	13 ^5,a^	17.5 ^2^	12.4 ^3^	14.8 ^3,a,b^	25.3 ^3^	17.5 ^3^	20.4 ^3,b^
Raw	0	0	0	0.4 ^6^	0.1 ^5^	0.4 ^5,a^	0	0.2 ^5^	0.2 ^6,a^	0.4 ^3^	0	0.4 ^4,a^	0	0	0

* *p* < 0.05, women vs. men. Different number superscripts show statistical differences (*p* < 0.05) within the same column. Different letter subscripts show statistical differences (*p* < 0.05) between age groups. Non-respondents: *n* = 15.

**Table 5:** Ways of learning to cook, the main criteria when planning meals at home, and preferred cooking techniques according to body mass index.

	Underweight (*n* = 32)	Normal Weight (*n* = 1031)	Pre-Obese (*n* = 663)	Obese (*n* = 187)
Declared Ways of Learning How to Cook (%)				
Own experience	40.9 ^a,1^	42 ^a,1^	45.4 ^a,1^	43.5 ^a,1^
From family	50 ^a,1^	42.1 ^a,1^	40.4 ^a,1^	47.9 ^a,1^
Books/TV	4.5 ^a,2^	4.9 ^a,2^	2.8 ^a,2^	2.5 ^a,2^
Internet	0	2.8 ^a,3^	1.8 ^a,2,3^	0.8 ^a,2^
Cooking Courses	0	1.6 ^a,3^	1.5 ^a,2,3^	0
Other	4.5 ^a,2^	6.3 ^a,2^	7.6 ^a,2^	3.4 ^a,2^
Main Criteria When Planning Meals at Home (%)				
Own food preferences	51.8 ^a,1^	28 ^b,1^	34.2 ^c,1^	37.4 ^a,c,1^
Other people’s food preferences	59 ^a,1^	58.2 ^a,2^	55.6 ^a,2^	60.5 ^a,2^
Healthiness of menu	45.5 ^a,1^	49.9 ^a,3^	44.2 ^a,3^	41.8 ^a,1^
Price	18.5 ^a,2,3^	17.9 ^a,4^	24.4 ^a,4^	23 ^a,3^
Convenience	3.4 ^a,3^	13.2 ^a,5^	15.7 ^a,5^	9.8 ^a,4^
Nutritional balance	20 ^a,b,2^	21.1 ^a,4^	15.5 ^b,5^	17 ^a,b,3^
Following a special diet	9.3 ^a,3^	15.5 ^a,4,5^	17.1 ^a,5^	19.8 ^a,3,4^
Preference for Cooking Techniques (%)				
Grill	82.7 ^a,1^	64.3 ^b,1^	68.3 ^a,b,1^	67.3 ^a,b,1^
Oven	50.8 ^a,2^	42.6 ^a,2^	42 ^a,2^	37.1 ^a,2^
Steaming	22 ^a,3^	19.4 ^a,2^	23.6 ^a,3^	15.9 ^a,3^
Stew	36.9 ^a,2,3^	46.5 ^a,1,2^	50.9 ^a,4^	49.4 ^a,4^
Frying	39.7 ^a,2,3^	20 ^b,2^	25.8 ^a,3^	18.5 ^b,3^
Microwave	13.3 ^a,3^	11.2 ^a,3^	14.4 ^a,5^	11.4 ^a,3^
Raw	0	0.4 ^a,4^	0.3 ^a,6^	0

Different lettered superscripts show statistical differences (*p* < 0.05) within the same row; different numbered superscripts show statistical differences (*p* < 0.05) within the same column. Non-respondents: *n* = 761.

## Appendix A. Questionnaire on Cooking Habits

**A. SOCIODEMOGRAPHIC DATA**

**Q.1. Gender:**	**Code**
Male	1
Female	2

**Q.2. How old are you? Age:**	**Code**
18–30 years	1
31–49 years	2
50–64 years	3
65–75 years	4
>75 years	5

**Q.3. Where do you live?** **Province: ________________** **City/Village: _____________** **Habitat size**	**Code**
< 2000 inhabitants	1
2001–10,000 inhabitants	2
10,001–10,000 inhabitants	3
100,001–500,000 inhabitants	4
> 500,000 inhabitants	5

**Q.4. Nationality**	**Code**
Spanish	1
Other: ___________	2

**Q.5. Employment Status: Are you currently…?**	**Code**	**Q.5.1. For how long you have been unemployed?**
Self-employed	1	______________________
Working for others	2	
Housewife (domestic work)	3	
Unemployed (Go to Question Q.5.1)	4	
Student	5	
Retired	6	
Other: __________________________	7	

**B. COOKING HABITS**

**Q.6. Do you know how to cook?**	**Code**
Yes	1
No	0

**Q.7. From 1 to 10, how much do you like cooking? Being 1: “I don’t like it at all” and 10 “I like it very much”?**									
I don’t like it at all		I don’t like it		I do not like or dislike it		I like it		I like it very much	
1	2	3	4	5	6	7	8	9	10
									

**Q.8. Are you the person in charge of food preparation at home?**	**Code**
I am 100% in charge	1
I am 50% in charge	2
I am <50% in charge	3
I am in charge occasionally	4
I am not involved in cooking	5
If Q.8 = 1 or 2, go to Q.8 If Q.8 ≠ 1 or 2, you are finished.	

**Q.9. How did you learn to cook?**	**Code**
Practicing (self-taught)	1
From a family member	2
Other person taught me (friends, partner…)	3
Reading cooking-books and magazines	4
Watching cooking TV-shows	5
Using the Internet	6
Cooking courses	7
Other (specify)	8

**Q.10. For how many persons do you usually cook?**

**Q.11. How much time do you spend cooking (weekly)?**

**Q.12. When organizing meals, what do you usually take into account? (Multiple choice)**	**Code**
Own food preferences	1
Other people’s food preferences	2
Healthiness of menu	3
Price	4
Convenience	5
Nutritional balance	6
Following a special diet	7
Other (specify)	8

**Q.13. What is your preferred cooking method? (Multiple choice)**	**Code**
Grill	1
Oven	2
Steaming	3
Stew	4
Frying	5
Microwave	6
Other (specify)	7

**Q14. Which of the following devices do you have in your kitchen? (Multiple choice)**	**Code**
Oven	1
Microwave	2
Dishwasher machine	3
Ceramic hob	4
Fridge	5
Mixer	6
Coffee maker	7
Toaster	8
Grill	9
Cooking Robot	10
Barbecue	11
Other	12
